# Assessment of the risk of infection among Romanian physicians at the outbreak of the SARS CoV-2 pandemic

**DOI:** 10.1080/13814788.2021.1963434

**Published:** 2021-08-25

**Authors:** Valeria Herdea, Alina Duduciuc, Raluca Ghionaru, Laura Comnea, Luminita Păduraru, Smaranda Diaconescu

**Affiliations:** a Department of Family Medicine, Pharmacy, Science and Technology ‘George Emil Palade’, University of Medicine, Târgu Mureș, Romania; b Faculty of Communication & Public Relations, National University of Political Studies and Public Administration, Bucharest, Romania; c Department of Family Medicine, Romanian Association for Pediatric Education in Family Medicine [AREPMF], Bucharest, Romania; d Department of Medicine, University of Medicine and Pharmacy ‘Grigore T. Popa’, Iasi, Romania

**Keywords:** COVID-19 pandemic, SARS-CoV-2 infection, Romanian physicians, perceived threat

## Abstract

**Background:**

In March 2020, the WHO declared the SARS CoV-2 pandemic. This had an immediate and dramatic impact on Romanian physicians.

**Objectives:**

To analyse SARS-CoV-2 risk perception among Romanian physicians following the official WHO pandemic announcement.

**Methods:**

A questionnaire was sent to Romanian physicians (*n* = 319) between 13 and 27 of March 2020 to determine the perceived threat of exposure to SARS CoV-2 infection, the assessment COVID-19 sources of documentation, physicians’ access to personal protective equipment and the attitude towards a prospective vaccine against SARS CoV-2.

**Results:**

Confronted with a new and unknown disease, the lack of appropriate information regarding disease management, media pressure and the lack of protective equipment, physicians experiencing a highly stressful a period. We found a significant relationship between the perceived level of fear and the risk of infection with SARS CoV-2 among respondents. A relationship was also found between the perceived level of fear related to COVID-19 and the acceptance of future vaccines against SARS CoV-2. Our data show that doctors working in urban areas considered the medical research on COVID-19 as clearer than those working in rural locations did.

**Conclusion:**

Pandemic preparedness should focus on measures that make medical practice safe (supplies, working protocols, experience sharing with experts/colleagues from other countries).

KEY MESSAGES

- At the start of the pandemic, most Romanian healthcare professionals reported a significant level of stress and fear about COVID-19 infection.
- International medical pandemic protocols should be adjusted to country-specific profiles.
- Pandemic preparedness should focus on safer medical practice and better healthcare policies and strategies for the long term.

## Introduction

On 11 March 2020, the spread of the COVID-19 disease resulted in the World Health Organisation (WHO) declaring a pandemic [1]. As scientific research emerged, it became apparent that this was a severe disease that would significantly impact globally [2–6]. To note a significant date of the evolution of the pandemic, at the midpoint of the last year, on 4 July 2020, 11,755,969 cases were confirmed, with a 7.58% death rate [7]. Those working as health care professionals (HCPs) had an increased risk of infection, with 3267 (14%) Romanian HCPs infected by June 2020 [8]. COVID-19 infection was defined as ‘the first new occupational disease to be described in this decade’ according to the Society of Occupational Medicine [9].

Previous studies exploring the psychological impact during previous epidemics (e.g. HIV, EBOLA, 2003–2005 Asian SARS epidemic) have shown that HCPs involved in the battle against communicable diseases experienced a degree of emotional overload triggered by the perceived risk of infection or by spread of the disease to those close to them. HCPs experienced anxiety, depression or burnout syndrome when their colleagues became their patients or had to overcome the stressful pressure to help others, to protect themselves and their relatives [10–15].

The first COVID-19 cases were confirmed in Romania on 22 February 2020. At that time, the Romanian medical system was confronted with a large number of SARS CoV-2 infections. In Romania, 747,592 tests were carried out, the number of totally infected people were 28,166 confirmed cases with 1708 deaths, 741 quarantined and 58,991 isolated persons until 3 July 2020. At the end of July 2020, 77.3% of those who died were over 60 years old and 59% of deaths were men [16].

On 7 April 2020, the first medical staff death due to SARS CoV-2 infection was registered in Romania; 14 deaths among Romanian HCPs were registered by July 2020. In Romania, until July 2020, one in eight people infected with the COVID-19 was a medical professional [17]. Compared to Romania (14.8% in April), other EU countries reported lower percentages for their infected HCPs at the pandemic peak (e.g. Spain 12% and Italy 9%) [18, 19].

Romanian medical staff have raised various concerns regarding the use of efficient protective measures, as well as the lack of clear protocols for assessing and caring for the patient with COVID-19 symptomatology. Consequently, we initiated and surveyed to assess the perceived risk of infection among the Romanian physicians.

## Methods

### Study design and participants

This research was conducted in conjunction with The Romanian Association for Paediatric Education in Family Medicine (AREPMF). The survey was conducted online, inviting a sample of 370 Romanian physicians from both urban and rural areas by email. The email informed the invited physicians about the aim of the study, participant anonymity and the publication of the results in a scientific journal. Before opening the link with the survey, participants were informed that study participation was optional and that completing the questionnaire represented their agreement to participate in the study. A link to the questionnaire was then provided (Google form).

The respondents were included in the sample according to eligibility criteria (inclusion criteria): participants confirmed as medical doctors; family physicians practicing in Romania and agreement with participation in the study. The exclusion criteria were: other professional categories (e.g. nurses and psychologists); physicians working overseas and refusal to participate in the study.

### Ethics

The survey complied with the Romanian legislation (Law 190/2018) and GDPR – the General Data Protection Regulation 679/2016. The study was conducted with the approval of the Ethics Committee of the AREPMF (no. 17 SNI/12.03.2020).

### Instrument design

A questionnaire was developed following consultations with clinical and research experts (physicians and sociologists). The draft questionnaire was pre-tested on a small sample of doctors (*n* = 30) excluded from the main study. The final version of the questionnaire (Supplemental material no. 1) consisted of 27 closed questions and one open question. The closed questions established socio-demographic data (gender, age, residence, office residence, years of experience in practicing of medicine) and variables including perceived risk of exposure to infection with SARS- CoV-2 (Likert scale), sources of information on COVID-19, perception of the clarity of the official information (Romanian authorities) and medical sources (research and clinical practice) on SARS-CoV-2 (Likert scale). The questionnaire also surveyed what information was needed for medical practice, level of fear of infection with SARS CoV-2 (afraid, slightly afraid, not afraid), level of medical equipment available, interaction with suspected patients and the likelihood of vaccination against SARS-CoV-2 in the future (agree, disagree, I will think about it). An open question was asked for the total number of infected Covid-19 patients in the physician’s practice.

### Statistical analysis

A descriptive data analysis was conducted. Fisher’s exact test was used to analyse the relationship between the perceived risk of infection with SARS CoV-2 among the medical area specialty of the respondents as well as the association between the likelihood of vaccination among respondents and perceived risk of exposure on COVID-19 infection. The statistical data was complete using SPSS version 20.0.

## Results

### Socio-demographic data

Of the 370 study invitations sent, 319 doctors participated in the study (response rate 86%). Most (225; 70%) were family physicians, while 70 (22%) were hospital practitioners. Other respondents delivered ambulatory care (12; 4%) and private practitioners in different specialties (12; 4%). Most respondents were female (276; 87%). The median age was 47 (range 31–64 years). Respondents working (257; 80%) or living (285; 89%) in the urban area outnumbered those with a rural workplace (54; 17%) or residence (34; 11%) (Table 1).

**Table 1.** Socio-demographic characteristics of the sample (*n* = 319).

	Number	%
Gender		
Male	43	13
Female	276	87
Age		
18–30 years	20	6
31–45 years	119	37
46–64 years	161	51
65+	19	6
Office Residence		
missing data	11	3
Urban	254	80
Rural	54	17
Medical area speciality		
family medicine	225	70
Hospital	70	22
Ambulatory	12	4
Others	12	4
Total	319	100

### Perceived exposure to infection with SARS CoV-2

Most of our respondents (258; 81%) were concerned about SARS CoV-2 infection. 191 (60%) physicians considered that they could be highly exposed to the infection while 67 (21%) reported that they could deal with medium exposure risk. In contrast, a fifth (20; 19%) of the respondents believed that they were safe (6%) or very safe (13%). Most of our respondents perceived a high level of exposure to SARS CoV-2, regardless of their medical area of specialty: 65% (*n* = 147) of the GPs and 18% (*n* = 40) of the hospital physicians were ‘very exposed and exposed’ whereas 44% (*n* = 31 GPs) and 35% (*n* = 25) hospital physicians answered ‘safe and very safe’ (Table 2).

**Table 2.** Assessment of the risk of infection among respondents (*n* = 319).

According to you, how much are you currently exposed to Covid-19 risk infection?	Number	%
Extremely exposed	191	60
Exposed	67	21
Moderately exposed	41	13
Safe	8	3
Very safe	12	4
Total	319	100

## Level of fear and perceived threat of exposure to SARS CoV-2 infection

The data revealed that one third (105; 33%) of respondents were afraid of the pandemic, with 193 (61%) moderately afraid. Twenty-three (7%) reported being not afraid. At the pandemic onset 40 (12.5%) respondents felt extremely exposed and unprotected when they needed to be in close contact with a patient suspected of being infected with SARS CoV-2. As the data show (Table 3), those respondents reporting a high level of fear are among the ones that perceived high risk of infection with SARS CoV-2 (*p* = 0.048).

**Table 3.** Relation between fear and risk perception^a^.

According to you, how much are you currently at risk for a Covid-19 infection?	How do you feel right now about the Covid-19 pandemic?			*p*-value*
	*afraid*	*not afraid*	*N cases*	
*exposed*	92 (87%)	14 (13%)	106 (100%)	0.048
*safe*	6 (60%)	4 (40%)	10 (100%)	
*N cases*	98 (86%)	18 (15%)	116 (100%)	

^a^ We performed a cross data type 2 × 2 between fear perception and risk exposure to arrive at Fisher exact test. We set aside those respondents that chose ‘slightly afraid’ (*n* = 191). It resulted in 127 respondents, 11 of 127 were moderately exposed (*n* = 11). It resulted in 116 respondents (cases) for Fisher exact correlation test.

*Fisher’s Exact Test.

### Ambiguity versus certainty of COVID-19 information

More than half of respondents (186; 58%) considered the information provided on the clinical manifestation of the SARS CoV-2 infection to be very confusing (57; 18%) and confusing (129; 40%).

### Assessment of medical information delivered by the medical sources on COVID-19

The information provided by medical sources (research and clinical practice) were assessed as unreliable by almost half of respondents (150; 47%), with 107 (33%) considering it ambiguous and 43 (14%) very ambiguous. Only 17% (*n* = 54) of the respondents assessed the medical information on Covid-19 as very clear (*n* = 9) and clear (*n* = 45). The results also show (Table 4) that those HCPs working in urban areas were more likely to consider medical research on COVID-19 as clearer than those with rural office residence (*p* = 0.019). Family medicine doctors report that medical research is more apprehensible compared with other doctors in this study. This was also the case for information provided by public health authorities (Table 4).

**Table 4.** Assessment of medical (scientific research & medical practice) and health public information on Covid-19 across physicians’ office residence and medical speciality area^a^.

	Ambiguous	Clear	*N* cases	*p*-value*
	Assessment of medical sources (scientific research & medical practice)			
Office residence				
Urban	126 (78%)	36 (22%)	162 (100%)	0.019
Rural	20 (59%)	14 (41%)	34 (100%)	
*N* cases	150 (74%)	54 (26%)	204 (100%)	
Medical area speciality				
Family medicine	93 (68%)	46 (33%)	139 (100%)	0.014
Hospital	40 (85%)	7 (15%)	47 (100%)	
Ambulatory	7 (100%)	0 (0%)	7 (100%)	
Others	10 (91%)	1 (9%)	11 (100%)	
*N* cases	150 (74%)	54 (26%)	204 (100%)	
	Assessment of health public information (from medical authorities)			
Medical area speciality				
Family medicine	69 (48%)	75 (52%)	144 (100%)	0.016
Hospital	29 (69%)	13 (31%)	42 (100%)	
Ambulatory	4 (80%)	1 (20%)	5 (100%)	
Others	8 (80%)	2 (20%)	10 (100%)	
*N* cases	110 (55%)	91 (45%)	201 (100%)	

^a^We performed a cross data type 2x3 (assessment of medical sources and medical area speciality) and 2 × 2 (for assessment of public health information and office residence). Our purpose was to arrive at Fisher exact test. We set aside those respondents that chose ‘not ambiguous, neither clear’ (*n* = 115). It resulted in 204 respondents for Fisher exact test.

*Fisher’s Exact Test.

### Likelihood of the vaccination against COVID-19 in the future

Two-thirds of respondents (196; 61%) would agree to vaccination while only 6 (2%) of them would not agree; 86 (27%) respondents would hesitate to accept a new vaccine. Those respondents who reported a high level of fear related to COVID-19 pandemic (Table 5) were more likely to accept vaccination than their counterparts (*p* = 0.031) (Table 6).

**Table 5.** The likelihood of vaccination among the sample (*n* = 319).

If a vaccine against COVID-19 infection will be available in the future, you would rather	*n*	*%*
Accept vaccination	195	61%
Refuse vaccination	6	2%
I will think about it	86	27%
Missing	32	10%
Total	319	100%

**Table 6.** Likelihood of vaccination according to fear perceived^a^.

If a vaccine against Covid-19 infection will be available in the future, you would rather	How do you feel right now about the Covid-19 pandemic?			*p*-value*
	*afraid*	*not afraid*	*n* *cases*	
*Accept vaccination*	76 (78%)	12 (55%)	88 (100%)	0.031
*Refuse vaccination*	21 (22%)	10 (45%)	31 (100%)	
*n* *cases*	97 (100%)	22 (100%)	119 (100%)	

^a^We excluded from the analyses those respondents (*n* = 86) saying they hesitate on vaccination (*I will think about it*). Then we looked at how the variable ‘likelihood of vaccination’ was distributed among a subsample (*n* = 119) composed of those that were affraid (*n* = 97) of covid-19 Pandemic and those that were unafraid (*n* = 22). The *p*-value resulted from the two-way Fisher exact test was statistically significant.

*Fisher’s Exact Test.

### Lack of personal protective equipment (PPE)

At questionnaire completion, 37% (118) of respondents had access to protective gloves, 61% (194) HCPs had access to a face mask, 1% (4) had access to overalls, and 1% (4) had access to protective eyewear.

Regarding the open question that referred to the total number of infected Covid-19 patients, we obtained a high rate of non-response. We assumed that most doctors did not complete this question because of lack of Covid-19 tests, data and screening capacity. Therefore, this question was excluded from the subsequent analysis.

## Discussion

### Main findings

This study assessed Romanian doctors’ immediate response to the COVID-19 pandemic and provided an insight into their perceptions and concerns at the start of the crisis. In this study, more than 60% of respondents report fear of being infected. They report a lack of clear medical information and working protocols and a shortage of PPE, which are likely to have contributed to their concerns.

### Interpretation

Whilst data is available on other infectious diseases [9–15], little is currently known about COVID-19.

In this study, more than 60% of respondents report fear of being infected. They report a lack of clear medical information and working protocols and a shortage of PPE, which are likely to have contributed to their concerns. Our findings are comparable to the experiences reported by HCP from China, Taiwan and Japan during the early 2003–2004 SARS epidemic [9–15].

Physicians increasingly practice evidence-based medicine (EBM), which provides a structured and systematic approach to managing critical clinical problems. Unfortunately, the rapid spread SARS CoV-2 infection has exceeded the capacity of medical systems to respond and has resulted in a lack of an appropriate evidence base to support doctors providing clinical care. The lack of access to complete scientific information, frequent changes to case definitions and population expectations have put significant pressure on medical personnel [20, 21]. Developing evidence-based guidelines and policy during pandemic situations will increase medical staff preparedness [10–14].

The Romanian medical staff encountered many obstacles, including changing disease management and multiple unpaid tasks (including updating outdated software programmes, providing statistical reports and arranging medical leave for patients) [20–22]. Such situations have been reported elsewhere that resulted in dissatisfaction among HCPs [23].

At the pandemic onset, PPE and disinfectants were not provided for medical staff. This, along with rising cases and fear amongst the workforce, resulted in medical staff resigning or retiring [23].

Despite scientific evidence regarding the benefits of general immunisation, misconceptions and mistrust of vaccine’s are found in Romania to result in low levels of vaccine uptake, despite significant death for conditions such as influenza [24–25]. This persists in the medical profession, with many respondents not wanting to take COVID-19 vaccination should it become available [26, 27].

### Strength and limitations

There are several limitations to this study, including the relatively small numbers of responders and the homogeneity of respondents. A more diverse sample should be selected to assess risk perception among Romanian HCPs. This study was also conducted early in the pandemic; a repeat survey may reveal different responses to some areas investigated as time progressed.

## Conclusion

Confronted with an unknown disease, the lack of appropriate information regarding disease management and limited access to personal protective equipment, HCPs have been facing a stressful period. This can inform future policy, allowing us to learn from past mistakes, providing better patient care and support for front line health professionals.

## Supplementary Material

Supplemental Material: Survey
