# Supplementary material for: Assessment of the risk of infection among Romanian physicians at the outbreak of the SARS CoV-2 pandemic
Source: Eur J Gen Pract. 2021 Aug 25;27(1):235–40. doi: 10.1080/13814788.2021.1963434 (PMC8405117; doi:10.1080/13814788.2021.1963434)
Supplement: Supplemental Material: Survey [file IGEN_A_1963434_SM0373.docx]

**Survey**

**Perception of risk among Romanian physicians at the outbreak of the COVID-19 pandemic**

Distinguished colleagues,

We kindly ask you to complete the survey below.

Goal: Analysing the way in which the risk of infection with SARS CoV-2 is perceived by the professional medical staff in Romania.

The survey is anonymous. We do not require your personal data by any means. The results of the survey will be published in a scientific journal and disseminated at academic conferences.

Completing this survey is optional. Completing this survey represents your agreement.

The study has the approval of the Ethics Committee of the AREPMF [no. 17 SNI/12.03.2020].

Optional: The survey participants can access the data by an e-mail enquiry ([secretariat@arepmf.ro](mailto:secretariat@arepmf.ro))

Thank you for your time!

1. Age:
   1. 18-30 yrs.
   2. 31-45 yrs.
   3. 46-64 yrs.
   4. > 65 yrs.

1A. Your working domain is:

1. Family Medicine
2. Ambulatory
3. Hospital
4. Others: ………………………………………………………………………….
5. Gender:
   1. M
   2. F
   3. Other: ……………………………………………………………………….
6. Seniority in the field:
   1. < 5 yrs.
   2. 6-15 yrs.
   3. 16-30 yrs.
   4. > 30 yrs.
7. Which area do you practice in?
   1. Urban
   2. Rural
   3. Both
8. Place of Residence:
   1. Urban
   2. Rural
9. How do you get to work?
   1. Walking
   2. Bicycle
   3. Personal car
   4. Bus/Minibus
   5. Subway
   6. Train
10. From where did you hear the first time about the SARS CoV-2 infection?
    1. Medical literature
    2. TV/Radio/Printed Press
    3. Social media, shared by other medical staff
    4. Social media, shared by patients
    5. Colleagues
    6. Family/Friends
11. On a scale from 1 to 5, where 5 is very clear and 1 very ambiguous, we kindly ask you to mention how ambiguous or clear do you consider the information received from MEDICAL SOURCES at this point in time (data from scientific research & medical practice) on COVID-19:

very ambiguous

- 1. 1
  2. 2
  3. 3
  4. 4
  5. 5

very clear

1. How ambiguous or clear do you consider, at this point in time, the information received from the AUTHORITIES/INSTITUTIONS with responsibilities in the medical domain:

very ambiguous

- 1. 1
  2. 2
  3. 3
  4. 4
  5. 5

very clear

1. How ambiguous or clear do you consider, at this point in time, the information on the CLINICAL features of the COVID-19 infection:

very ambiguous

- 1. 1
  2. 2
  3. 3
  4. 4
  5. 5

very clear

1. During the last month, did you access medical articles, in order to learn more about COVID-19?
   1. Yes
   2. No
   3. I’m not interested in this subject
2. At this point in time, what kind of information do you need in order to understand the COVID-19 infection?
   1. Official data from the authorities
   2. Medical research & practical experience
3. What is your perception regarding the global spread risk of COVID-19 disease?
   1. Real risk
   2. Undervalued
   3. Overvalued
4. What is, from your perception, the spread risk of the COVID-19 infection in Romania?
   1. Real risk
   2. Undervalued
   3. Overvalued
5. Do you consider yourself exposed to being infected with SARS-CoV-2?

extremely exposed

- 1. 1
  2. 2
  3. 3
  4. 4
  5. 5

I feel safe

1. Choose the statement that best defines your way of thinking about the COVID-19 at this point in time:
   1. I can be infected.
   2. I can’t be infected.
   3. I can be infected, but I’m optimist that I will not.
   4. The others can be infected, but I’m resistant.
   5. I can develop a severe form of the disease.
   6. I can die from being infected with SARSCoV-2.
2. Choose the statement that best defines your perception of the global spread risk of COVID-19:
   1. The COVID-19 infection is rapidly growing.
   2. The COVID-19 infection is steadily growing.
   3. The COVID-19 infection is slowly growing.
3. The following statements are true or false?

| **Statement** | **False** | **True** | **I’m not sure** |
| --- | --- | --- | --- |
| The SARS-CoV-2 virus is created in a lab. |  |  |  |
| The virus source is unknown. |  |  |  |
| A vaccine will be developed until the next season. |  |  |  |
| COVID-19 will stop when the temperatures will be higher. |  |  |  |
| The elderly are more vulnerable to being infected with SARS CoV-2. |  |  |  |
| The infection can be treated with antivirals. |  |  |  |
| Children are not vulnerable to the SARS-CoV-2 infection. |  |  |  |
| The COVID-19 danger is being overstated by the media. |  |  |  |
| COVID-19 will not develop as bad as it is estimated. |  |  |  |

1. Choose the information that best suits your situation:
   1. I have access to the therapeutic schemes applied to the COVID-19 infection.
   2. I do not have access to the therapeutic schemes applied to the COVID-19 infection.
2. How are you feeling regarding the COVID-19 pandemic?
   1. I am afraid.
   2. I am somehow afraid.
   3. I am not afraid.
3. At this point in time, which parts do you have of your personal protective equipment?
   1. Protection mask
   2. Gloves
   3. Protective suit
   4. Protective glasses
4. From your perspective, how did the patients’ behaviour change from the moment of the expansion of the SARS CoV-2 infection?
   1. Patients are not coming to the doctor’s office.
   2. The patients are exaggerating the symptoms and solicit us without a reason.
   3. I have not noticed changes in their behaviour.
5. Do you consider, at this point in time, your patients trust you when you provide them information on the SARS CoV-2 infection?
   1. Yes
   2. No
6. Did you come into contact with patients suspected of being infected-SARS Cov-2/ COVID-19?
   1. Yes
   2. No
   3. I don’t know

24.A. If the answer to Q24 is “Yes”, please answer the following question: How did you perceive, on a personal level, the contact with a patient suspected of being infected with SARS CoV-2?

a. Exposed and unprotected.

b. Safe, it was like a normal contact with any other patient.

c. Nervous/unsure regarding the patient’s clinical evaluation.

1. From your point of view, when will the COVID-19 pandemic end?
   1. 6 months
   2. 1 year
   3. 2 years
   4. I don’t know
2. From the first confirmed case in Romania, how satisfied are you by the collaboration with the Health authorities/institutions?

Not satisfied at all

- 1. 1
  2. 2
  3. 3
  4. 4
  5. 5

Very satisfied

1. A vaccine will be developed in the future to protect us from the SARS-CoV-2 virus. When the vaccine will be available, you would rather
   1. Accept vaccination
   2. Refuse vaccination
   3. I will think about it
2. How many infected patients do you have till now?

-------------------------------------------------------------
